# Wogonin Suppresses IL-10 Production in B Cells via STAT3 and ERK Signaling Pathway

**DOI:** 10.1155/2020/3032425

**Published:** 2020-06-01

**Authors:** Li Fan, Dongbo Qiu, Guo Huang, Jingrou Chen, Qiongli Wu, Shiqiu Xiong, Changyou Wu, Yanwen Peng, Qi Zhang

**Affiliations:** ^1^The Biotherapy Center, The Third Affiliated Hospital, Sun Yat-sen University, Guangzhou 510630, China; ^2^Department of Immunology, Zhongshan School of Medicine, Sun Yat-sen University, Guangzhou 510080, China; ^3^Department of Rheumatology and Immunology, The Eighth Affiliated Hospital, Sun Yat-sen University, Shenzhen 518030, China; ^4^Cell Biology Group, National Measurement Lab, LGC, Fordham, Cambridgeshire, UK CB7 5WW

## Abstract

Wogonin (5,7-dihydroxy-8-methoxyflavone) is an ingredient of the extracts from Scutellaria baicalensis, which has documented a wide spectrum of anti-inflammatory and antitumor activities, including inhibiting regulatory T cells, regulating effector T cell functions, and mediating macrophage immunity. However, the potential effect of Wogonin on B cells has not been fully understood. Here, our results showed that Wogonin inhibited IL-10 secretion in B cells. When purified B cells were activated by lipopolysaccharide (LPS) in vitro, the amount of IL-10 production in supernatant was decreased by Wogonin significantly. The protective role of B cells on dextran sulfate sodium- (DSS-) induced colitis was alleviated after exposure to Wogonin. Furthermore, administration of Wogonin on LPS-treated B cells suppressed phosphorylation of STAT3 and ERK, but not AKT. Interestingly, among those IL-10 signaling-associated transcription factors, mRNA and protein levels of Hif-1*α* were specifically decreased by Wogonin. Overall, our study indicates that Wogonin suppresses potentially IL-10 production in B cells via inhibition of the STAT3 and ERK signaling pathway as well as inhibition of mRNA and protein levels of the transcription factor Hif-1*α*. These results provide novel and potential molecular targets of Wogonin in B cells and help us further understand its mechanism of action, which could potentially improve its clinical application in the future.

## 1. Introduction

Wogonin is a natural flavonoid component extracted from the dried root of Scutellaria baicalensis. Various studies showed its diverse immunoregulatory effects of anti-inflammatory [1, 2], anticancer [3], antivirus [4], and antiallergy [5]. Wogonin exerts immunoregulatory effects by influencing multiple immune cells, such as inhibiting regulatory T cell (Treg) activities [6, 7], modulating Th17 differentiation [8], and mediating macrophage immunity [9]. However, the potential effect of Wogonin on B cells is still unclear.

Conventionally, B cells were deemed as the humoral immune cells executing antigen presentation and antibody secretion. However, progressive proof displayed immunosuppressive cytokine production from B cells, such as IL-35, TGF-*β*, and IL-10. Further investigation defined several subpopulations of B cells retaining regulatory function, such as peripheral circulating CD19^+^CD1d^hi^CD5^+^, peritoneal CD5^+^B1a, CD19^+^CD23^hi^CD21^hi^IgM^+^, CD19^+^CD24^hi^CD38^hi^, CD19^+^ B220^low^, and CD19^+^CD138^+^ plasma cells. Although they have different phenotypes, development pathways, anatomical distribution, and pathogenic implications, they all possess an immunoregulatory activity, which put them onto center stage by basic and clinical scientists [10, 11].

Wogonin repelled the production of immunoglobulins and cytokines (such as IL-4, IL-5, and IL-10) from mesenteric lymphocytes in DSS-induced colitis in vivo [12]. The anti-inflammatory effect of Wogonin associated with its suppression the production of nitric oxide, cytokines, and chemokines in dsRNA-induced macrophages via the calcium-STAT pathway [13]. Dandawate et al. [6] also described the inhibition of glioma-induced, TGF-*β*1-mediated regulatory T cell activity by Wogonin in vitro. In this work, Wogonin inhibited the IL-10 secretion of regulatory T cells. Instrumentally, Wogonin repressed Smad-3, GSK-3*β*, and ERK1/2 signaling in regulatory T cells. Meanwhile, P38 phosphorylation was markedly boosted, implying that Wogonin may regulate T cells' function via Smad and non-Smad signaling pathways. However, whether and how Wogonin perturb the production of IL-10 in B cells are still unknown.

As one of the main effector cytokines from regulating immune cells, IL-10 was highlighted in different fields. Molecular machinery of IL-10 production has been investigated in details. The activation of the MAPK extracellular signal-regulated kinase (ERK) 1 and ERK 2 [14, 15], PI3K-AKT pathway [16, 17], and NF-*κ*B [18] during IL-10 production was documented in macrophages and mDCs. Furthermore, it was proved that activation of STAT3 regulated IL-10 production [19], which was critical for LPS-induced IL-10 gene expression [20]. TLR signaling also controlled potent secretion of IL-10 in human B cells via mediating the activation of STAT3 and ERK [21]. Meanwhile, several transcription factors were proved to be involved in the regulation of IL-10 gene expression, such as Foxp3, Gata3, aryl hydrocarbon receptor (Ahr), JunB, Egr2, and Batf and c-maf, which were demonstrated to bind IL-10 promoter and increase IL-10 expression in macrophages [22–27]. Interestingly, hypoxia-inducible factor 1-alpha (Hif-1*α*), the main hypoxia-initiated transcription regulator, was shown to drive IL-10 expression in CD1d^hi^CD5^+^ B cells, hence exerting their protective roles in autoimmune diseases [28].

In this study, we aimed to investigate the response and mechanism of Wogonin on IL-10 production of LPS-treated B cells. Our data suggested that Wogonin impeded IL-10 production from LPS-treated B cells in vitro and weakened the immunoregulatory effect of B cells on DSS-induced murine colitis. In further investigation in LPS-treated B cells, Wogonin was shown to constrain phosphorylation of ERK and STAT3 and Hif-1*α* transcription. Our studies suggested that Wogonin might inhibit IL-10 production from B cells via modulating Hif-1*α* and ERK, STAT3 signaling pathway, which would help us understand more fully the role of Wogonin in immune regulation.

## 2. Materials and Methods

### 2.1. Mice

Six to eight weeks male C57BL/6 mice were purchased from Model Animal Research Center of Nanjing University. Mice were bred under specific pathogen-free condition and received a 12 h light : 12 h dark cycle. All animal experiments were approved by the institutional review committee of the Sun Yat-sen University and performed in strict compliance with the national and institutional guidelines.

### 2.2. Cell Isolation, Enrichment, and Culture

The spleen was minced and passed through a 70 *μ*m nylon cell strainer; lymphocytes were isolated via Ficoll-Hypaque (Tianjin HaoYang Biological Manufacture, Tianjin, China). Cells were resuspended in completed RPMI-1640 medium (Gibco, Grand Island, USA) containing with 10% heat-inactivated fetal calf serum (Hyclone, Australia), 100 *μ*g/mL streptomycin, 100 U/mL penicillin, 2 mM L-glutamine, and 50 *μ*M 2-mercaptoethanol (Life Technologies). CD19^+^ cells were purified by negative selection nanobeads according to the manufacturer's protocols (BioLegend, San Diego, CA, USA). The purity of CD19^+^ cells was verified on FACS Arial II (BD Biosciences). 1 × 10^6^/mL purified B cells were cultured with/without 2 *μ*g/mL LPS (Sigma-Aldrich, St Louis, MO, USA) for 6 h, 12 h, 24 h, and 48 h in the presence of Wogonin (MedChemExpress, China) with different concentrations (0, 12.5, 25, and 50 *μ*M).

### 2.3. DSS-Induced Acute Colitis and Abdominal B Cell Adoptive Transfer

Mice were divided randomly into four groups, six in each group. 5% DSS (MP Biomedicals, USA) in drinking water was administered to induce acute colitis.

**Figure 1:**
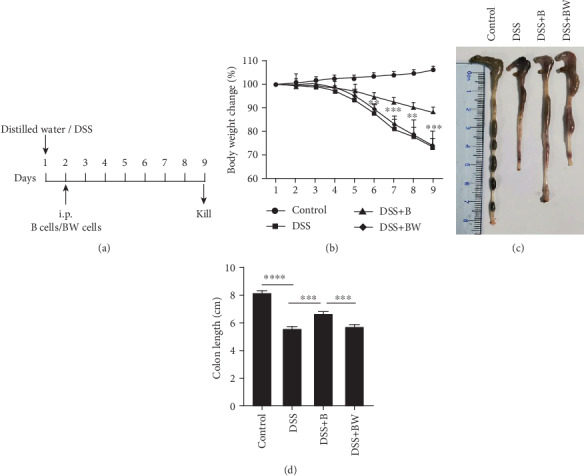
Effect of Wogonin on B cells in mouse with acute colitis induced by DSS. The drinking water containing 5% DSS was administered for one day, followed by intraperitoneal injection of B cells (DSS+B group) or Wogonin-pretreated B cell (DSS+BW group) on the second day and sacrificed on the ninth day. (a) The procedure of animal model in C57BL/6 mice. (b) The body weight changes of mice after DSS induction of colitis. ^∗∗^*P* < 0.01; ^∗∗∗^*P* < 0.001 for comparison with the DSS+B group. (c) Representative colonic length of mice was measured in four groups. (d) Quantification of colonic length of mice in four groups was shown. Data are presented as mean ± SD (*n* = 6 per group). ^∗∗∗^*P* < 0.001; ^∗∗∗∗^*P* < 0.0001.

Peritoneal cavity cells from mouse were isolated and resuspended in PBS according to a published protocol [29]. After being cultured for 2 h in the incubator, macrophages attached to the plate surface, suspension B cells were harvested in the supernatant. After being treated with/without 50 *μ*M Wogonin for 6 h, 2 × 10^6^ B cells were suspended in 200 *μ*L of PBS and transferred intraperitoneally into recipient mice on day 1 of colitis induction. Body weight was supervised every day. All mice were terminated on the ninth day. The colon length was measured to evaluate the colitis. Skeptical procedure was described in Figure 1(a).

### 2.4. Flow Cytometry for Phenotyping and Cytokine Secretion

Flow cytometry analysis for cell phenotype and intracellular cytokine secretion has been described previously [30]. Briefly, cells were washed twice and maintained in 100 *μ*L of PBS containing 0.1% BSA and 0.05% sodium azide. For phenotyping, cells were stained with corresponding Abs for 30 min at 4°C in the dark. For the analysis of intracellular cytokines, after surface staining as described above, cells were fixed with 4% paraformaldehyde and permeabilized in staining buffer containing 0.1% saponin (Sigma-Aldrich, St Louis, MO, USA) for at least 2 h or overnight at 4°C. After being washed twice, cells were incubated with the respective cytokine Abs for 30 min at 4°C. Samples were acquired on FACS Arial II, and data were analyzed by FlowJo software (Tree Star, San Carlos, CA, USA). The antibodies used are shown in Table 1.

### 2.5. Cell Viability Measurement

To avoid dead cell-induced false positive, cell viability of LPS-activated B cells was investigated. 2 × 10^6^/mL B cells were cultured in 96-well plates with or without LPS and Wogonin for 6 h, 12 h, 24 h, and 48 h. The cell viability was measured by a CCK8 kit (Beyotime, Shanghai, China) according to the supplier's instructions.

### 2.6. ELISA

Purified B cells were suspended in complete RPMI 1640 medium at a density of 1 × 10^6^/mL and stimulated with/without LPS and Wogonin for 6 h, 12 h, 24 h, and 48 h in 24-well plates. Cell-free supernatants were collected for the quantitative assessment of IL-10 according to the manufacturer's instructions (BioLegend, San Jose, CA, USA).

### 2.7. Histology

The distal colon tissues were fixed in formalin-free fixative and embedded by paraffin; tissues (5 *μ*m) were stained with hematoxylin and eosin according to standard techniques. The histological score was assessed by inflammation (I) (3, severe; 2, moderate; 1, slight; and 0, none); damage degree (D) (3, transmural; 2, mucosa and submucosa; 1, mucosa; and 0, none); regenerative ability (R) (4, no tissue repair; 3, surface epithelium not intact; 2, regeneration with crypt's depletion; 1, almost complete regeneration; and 0, complete regeneration or normal tissue); crypt lesion (C) (4, entire crypt and epithelium lost; 3, only surface epithelium intact; 2, basal 2/3 damaged; 1, basal 1/3 damaged; and 0, none); and percentage (P) (4, 76%–100%; 3, 51%–75%; 2, 26%–50%; and 1, 1%–25%) following the previous reports [31]. The histology score was calculated by I + D + R + C + P and evaluated by an experienced pathologist.

### 2.8. Western Blot

CD19^+^ B cells were challenged by LPS for 6 h with/without Wogonin (12.5, 25, and 50 *μ*M). Cells were lysed in RIPA cell buffer (Beyotime) supplemented with PMSF (Solarbio) and protease inhibitors (Roche) on ice for 30 min. 50 *μ*g of protein sample in the lysate was separated in 10% SDS-PAGE gels and transferred to PVDF membranes (Millipore). Membranes were blocked in TBST (Triton-containing Tris-buffered saline) with 5% milk for 2 h at room temperature and then incubated with specific primary antibodies overnight at 4°C. The secondary antibodies were added to the membranes and incubated for 1 h. Blot bands were visualized by enhanced chemiluminescence. The quantitative analysis was evaluated using ImageJ software according to the grey value of western blot bands.

The primary antibodies were used including Phospho-STAT3 (p-STAT3), total STAT3 (t-STAT3), Phospho-AKT (p-AKT), total AKT (t-AKT), Phospho-ERK (p-ERK), total ERK (t-ERK), Hif-1*α* (all were from Cell Signaling Technology), and GAPDH (Santa Cruz Biotechnology). The secondary antibodies were also purchased from Cell Signaling Technology.

### 2.9. Real-Time PCR Analysis

To analyze the gene transcription, beads purified and purity validated CD19^+^ B cells were cultured with or without LPS along with Wogonin (0, 12.5, 25, and 50 *μ*M) for 6 h as before. Total RNA was extracted by Trizol reagent (Invitrogen) and then reverse-transcribed with a cDNA Synthesis Supermix (Novoprotein Scientific Inc.) according to the manufacturer's instruction. The mRNA amount of Foxp3, c-maf, JunB, Gata3, Egr2, Ahr, Hif-1*α*, and Batf was assessed via SYBR Green probes (Novoprotein Scientific Inc.) and Step One Plus™ Real-Time PCR System. The data were calculated using the 2^-*ΔΔ*CT^ method. Specific primer sequences are listed in Table 2.

### 2.10. Statistical Analysis

All statistical analysis was presented by GraphPad Prism 5 and 6 (GraphPad Software Inc.). Statistical significance was analyzed by unpaired Student's *t* test (two groups) or one-way ANOVA (more than two groups). Results were shown as mean ± SD. ^∗∗∗∗^*P* < 0.0001, ^∗∗∗^*P* < 0.001, ^∗∗^*P* < 0.01, and ^∗^*P* < 0.05.

## 3. Results

### 3.1. Effect of Wogonin on the Production of IL-10 in B Cells

**Figure 2:**
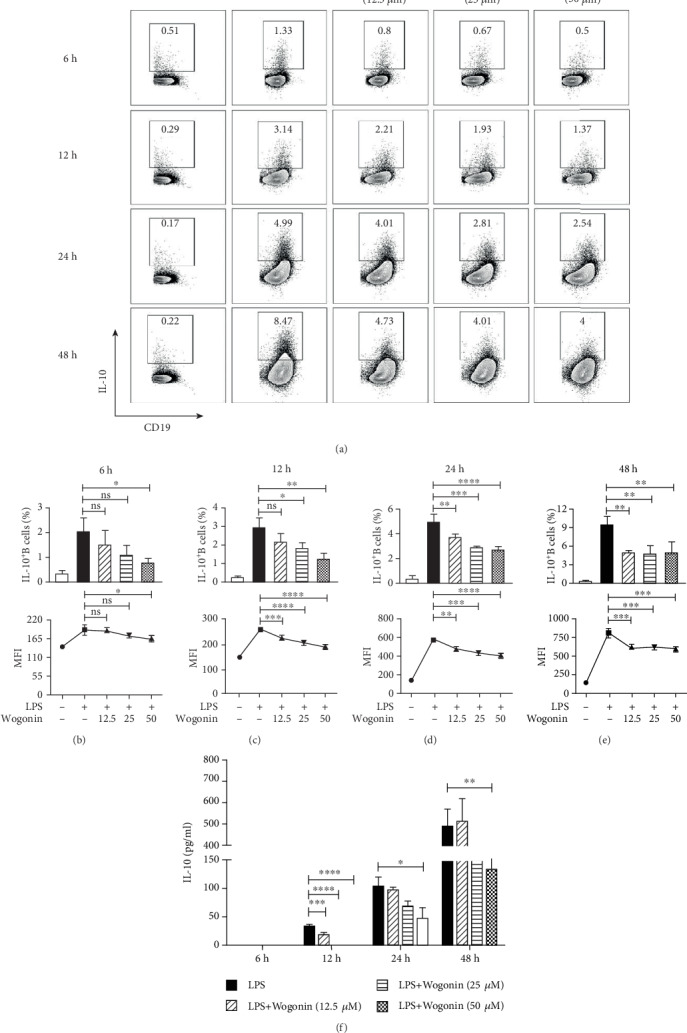
Wogonin suppressed the production of IL-10 in LPS-induced B cells. The purified CD19^+^ cells from the spleen were stimulated with or without LPS in the presence or absence of Wogonin (0, 12.5, 25, 50, and 100 *μ*M) for 6 h, 12 h, 24 h, and 48 h, and the frequencies of IL-10-producing B cells were analyzed. (a) Representative plots of IL-10-producing B cells with LPS induced in the presence or absence of Wogonin. (b–e) Quantification and MFI of IL-10-producing B cells at 6 h, 12 h, 24 h, and 48 h, respectively. (f) The supernatants from the different culture conditions were assayed by ELISA for the production of IL-10. Data are expressed as mean ± SD of three independent experiments and compared with one-way ANOVA. ^∗^*P* < 0.05; ^∗∗^*P* < 0.01; ^∗∗∗^*P* < 0.001; ^∗∗∗∗^*P* < 0.0001; ns: no significance.

Previous studies have reported that Wogonin can effectively promote the apoptosis of various cancer cells without cytotoxicity to other normal cells in the safe concentration range (10-100 *μ*M) [32–34]. To explore the effect of Wogonin on B cells, the purified CD19^+^ B cells from the spleen were stimulated with/without LPS along with Wogonin (0, 12.5, 25, 50, and 100 *μ*M) for 6 h, 12 h, 24 h, and 48 h (the purity of CD19^+^cells was shown in Figure S1). Since 100 *μ*M Wogonin induced significant cell death, 12.5, 25, and 50 *μ*M Wogonin were applied in the subsequent studies (Figure S2). The production of IL-10 from B cells under different concentrations of Wogonin was assessed dynamically. After 12 to 48 h treatment, 25 and 50 *μ*M Wogonin suppressed IL-10 production from B cells significantly; 12.5 *μ*M Wogonin showed similar suppression under 24 h and 48 h administration. During 12 h and 24 h treatment, Wogonin showed dose-effect on IL-10 production. After 48 h Wogonin treatment, all 3 concentrations of Wogonin showed a similar effect on B cells, which indicated their thresholds (Figures 2(a)–2(e)). The secreted IL-10 amount in the supernatant was assessed as well, which was compatible with intracellular quantity (Figure 2(f)).

### 3.2. Effect of Wogonin on the Surface Molecules of B Cells

**Figure 3:**
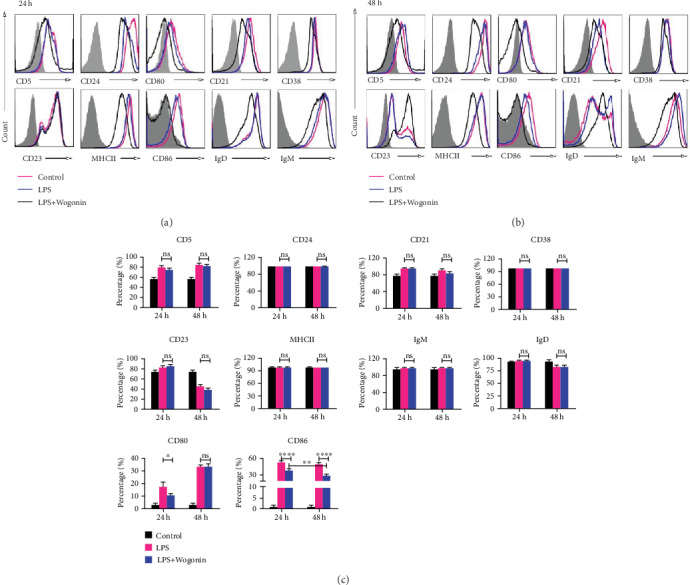
Effect of Wogonin on the surface molecules of B cells. CD19^+^ cells were cultured with LPS in the presence of 12.5 *μ*M Wogonin for 24 h and 48 h to analyze the surface marker levels. (a, b) The expression levels of CD5, CD24, CD21, CD38, CD23, MHCII, IgM, IgD, CD80, and CD86 in untreated B cells (black), LPS-treated B cells (red), and LPS+Wogonin-treated B cells (blue) were measured by FACS, respectively. (c) Quantification of B cell surface molecules. Data are expressed as mean ± SD of three independent experiments. ^∗^*P* < 0.05; ^∗∗^*P* < 0.01; ^∗∗∗∗^*P* < 0.0001; ns: no significance.

After investigation on IL-10 secretion, the phenotype of B cells was also assessed under different conditions of Wogonin administration. Frequencies of typical B cell markers, such as CD5, CD24, CD21, CD38, CD23, MHCII, IgD, IgM, CD80, and CD86, were analyzed by flow cytometry. We found that the expression amount of most surface markers did not obviously change by Wogonin (Figure S3); only frequencies of CD80 and CD86 were significantly decreased by Wogonin after LPS stimulation (Figures 3(a)–3(c)). These observations indicated that Wogonin might regulate antigen presentation capability of B cells, which could be interesting for immunotherapy of PD-1/PDL-1 Ab in different clinical settings.

### 3.3. Effect of Wogonin on B Cells in Mouse with Acute Colitis

To validate our observations in vitro, the response of B cells to Wogonin challenge was evaluated in vivo. Isolated B cells from mouse peritoneal cavity were challenged with/without Wogonin, and then, their impingement on DSS-induced colitis was examined. As shown in Figure 1(a), the body weights of DSS-treated mice were significantly decreased from day 5, whereas intraperitoneal injection of B cells significantly attenuated the loss of body weight in comparison with the DSS group, which suggested the immunological regulation of adoptive transferred B cells, and this regulation function was lost in Wogonin-treated B cells (Figure 1(b)). Colon length was assessed among these 4 groups of mice, which echoed weight loss (Figures 1(c) and 1(d)). These results suggested that Wogonin treatment abrogated immunological regulation of B cells in vivo.

**Figure 4:**
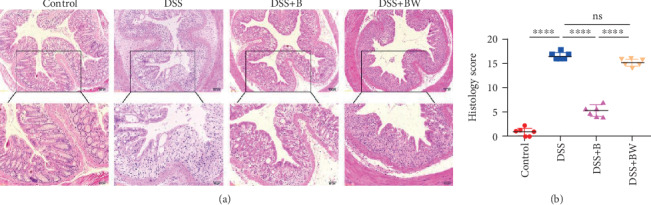
Effect of Wogonin on colon damage in mice induced by DSS. (a) Colon sections were stained with hematoxylin and eosin (H&E) (magnification ×100/200). (b) Histological score of colon tissues was assessed (*n* = 6 per group). ^∗∗∗∗^*P* < 0.0001; ns: no significance.

To further verify the role of Wogonin on adoptive transferred B cells in vivo, in situ histopathological analysis of colon tissues was investigated among all 4 groups of animals. Inflammation, mucosal and submucosa damage degree, epithelial intact, distortion of crypts, and percentage were compiled into histology score. Similar to weight loss and colon loss, significant colon damage caused by DSS administration was attenuated by transferred B cells, but this debilitation was suppressed by Wogonin treatment (Figures 4(a) and 4(b)).

### 3.4. Effect of Wogonin on the STAT3 and ERK Signaling Pathway of LPS-Mediated B Cells

**Figure 5:**
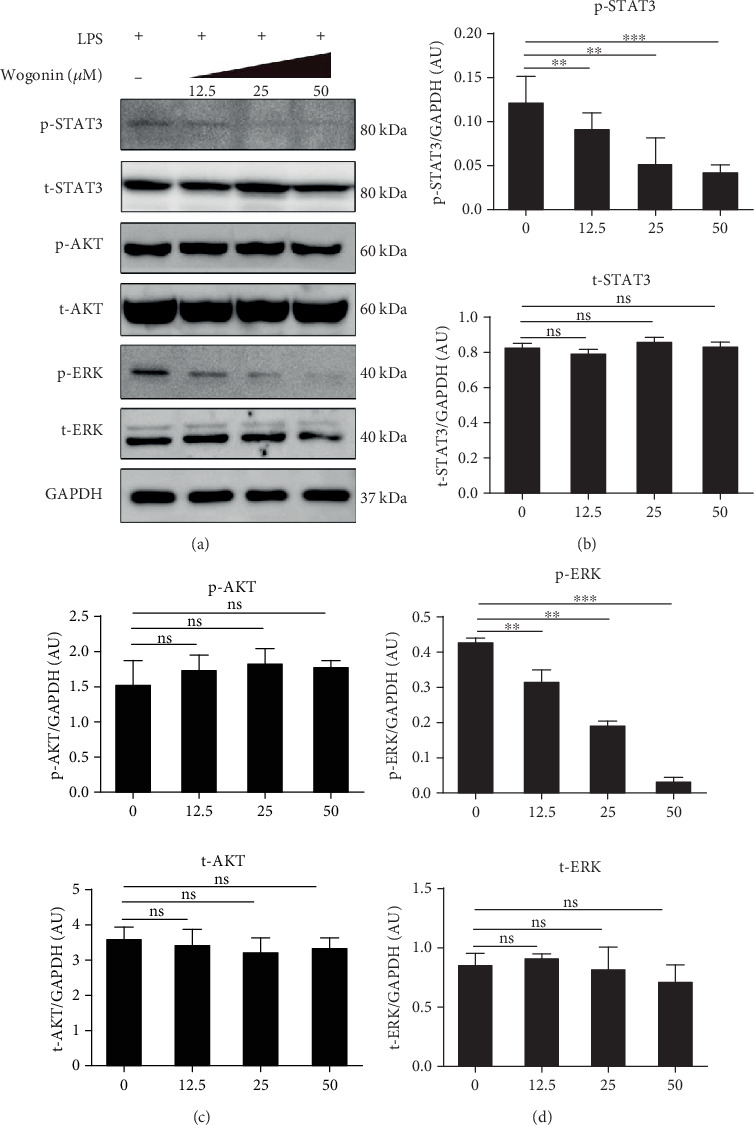
Dose-dependent inhibition of STAT3 and ERK phosphorylation by Wogonin in LPS-treated B cells. CD19^+^ B cells were cultured with LPS for 6 h in the presence or absence of Wogonin (12.5, 25, and 50 *μ*M). (a–d) The total and phosphorylated protein levels of STAT3, AKT, and ERK in LPS-treated B cells under different concentrations of Wogonin were analyzed and quantified by western blot. Data are representative of five independent experiments. GAPDH was used as loading controls. ^∗∗^*P* < 0.01; ^∗∗∗^*P* < 0.001; ns: no significance.

To explore the potential molecular mechanisms of Wogonin impinge on B cells, we assessed signaling pathways involved in IL-10 production. Strikingly, phosphorylation of STAT3 and ERK was decreased via Wogonin treatment, and this suppression showed dose-effect tendency. On the contrary, AKT phosphorylation was kept intact on Wogonin treatment (Figures 5(a)–5(d)). This investigation strongly suggested Wogonin suppression on IL-10 production from B cells involved in STAT3/ERK signaling pathway, instead of AKT/ERK.

### 3.5. Wogonin Declined Hif-1*α* Transcription Specifically

**Figure 6:**
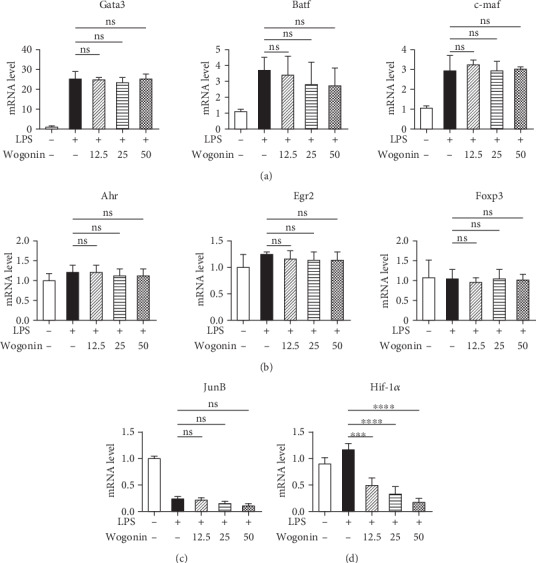
Effect of Wogonin on the relative transcription factor levels of IL-10 in B cells. B cells were cultured with or without LPS in the presence of Wogonin (0, 12.5, 25, and 50 *μ*M) for 6 h. Total RNA was extracted by Trizol and then reverse-transcribed. (a–d) The mRNA levels of Gata3, Batf, c-maf, Ahr, Egr2, Foxp3, JunB, and Hif-1*α* were measured by RT-PCR. The results are representative of three repeated experiments. ^∗∗∗^*P* < 0.001; ^∗∗∗∗^*P* < 0.0001; ns: no significance.

To further explore other potential mechanisms of Wogonin-mediated inhibition of the IL-10 production in B cells, the mRNA amount of transcription factors regulating IL-10 expression in B cells was investigated via RT-PCR. Among Foxp3, c-maf, JunB, Gata3, Egr2, Ahr, and Batf, Hif-1*α* was the only transcription factor whose mRNA amount was significantly abrogated by Wogonin treatment, and this suppression showed a dose-effect curve (Figures 6(a)–6(d)).

**Figure 7:**
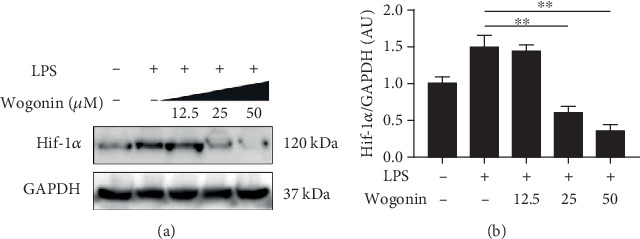
Wogonin decreased the Hif-1*α* expression in LPS-treated B cells. Purified B cells were cultured with LPS for 6 h in the presence of different concentrations of Wogonin (0, 12.5, 25, and 50 *μ*M). (a) Hif-1*α* amount from LPS- and Wogonin-treated B cells was assessed by immunoblots; GAPDH was applied as loading control. (b) The quantitative grey intensity of Hif-1*α* blots from three independent experiments was shown. ^∗∗^ *P* < 0.01.

In order to verify the change of Hif-1*α* caused by Wogonin, Hif-1*α* protein expression was also assessed by western blot. As expected, Wogonin significantly decreased the protein level of Hif-1*α* in B cells after LPS stimulation in a dose-dependent manner, which was consistent with the change of mRNA level (Figures 7(a) and 7(b)). Hence, our data suggested that Hif-1*α* was a potential target of Wogonin in B cells.

## 4. Discussion

Wogonin is a flavonoid-like compound, purified from Scutellaria baicalensis. Originally, Wogonin showed anxiolytic properties in mice [35]. Later on, extensive investigations on different tumor models proved the therapeutic effect of Wogonin [36], which is due to their regulation in different signaling pathways [37].

Recently, the anti-inflammatory and immune regulatory effect from Wogonin was also documented [7, 38]. Wogonin shaped phenotype and function of macrophage, effector and regulatory T cells via different signaling pathway. The progress indicated comprehensive clinical implications of this compound and drew broad attention on this extract.

Paralleling to Treg, a small subset of B cells participating in immunomodulation of immune responses has been described in the models of inflammation, autoimmune diseases, transplantation, and antitumor immunity. IL-10 from Breg is one of the main regulatory molecules in these pathogenic processes. Hence, we investigated the effect of Wogonin in B cells, focusing on IL-10 production. To our knowledge, this is the first report on this topic.

TLR agonists including LPS (TLR4), R848 (TLR7, 8), or CpG (TLR9) can potently activate B cells and induce IL-10 production [39]. Therefore, we observed the frequency of IL-10 production of B cells stimulated by LPS before and after Wogonin treatment. In an in vitro model, Wogonin significantly decreased the IL-10 secretion of B cells. Interestingly, Wogonin was also shown to inhibit IL-10 secretion from regulatory T cells specifically [6]. Furthermore, the protective role of B cells on DSS-induced colitis was alleviated after exposure to Wogonin in vivo. These results indicated that Wogonin executed regulatory effect on B cells via inhibition of IL-10 secretion.

To validate the specificity of Wogonin on B cells, we investigated the consequence of Wogonin on B cells' phenotype. The intact amount of CD5, CD24, CD21, CD38, CD23, MHCII, IgD, and IgM on B cells verified the tolerance of B cells under current experimental conditions, which highlighted the specificity of Wogonin on IL-10 secretion from B cells. Moderately, Wogonin decreased the frequencies of CD80 and CD86. At the moment, it is difficult to determine that this change was due to direct implementation of Wogonin on B cells or their regulation via monocytes/macrophage. However, the influence of Wogonin on these checkpoint molecules indicated potential preclinical combinative regime.

Mechanically, TLR4 signaling switched on IL-10 via both ERK and AKT pathways. Previous studies also have shown that the activation of STAT3 and ERK is required for TLR-induced IL-10 production in human B cells [21]. However, our data showed that Wogonin treatment diminished STAT3/ERK signaling only, it did not abate AKT phosphorylation under current conditions. On the contrary, Parajuli et al. [40] reported that Scutellaria flavonoids could inhibit the phosphorylation of Akt and GSK-3*β* in malignant gliomas. Further investigation may uncover a novel target of Wogonin in B cells.

Since Foxp3, c-maf, JunB, Gata3, Egr2, Ahr, Hif-1*α*, and Batf were identified as the main transcription factors regulating IL-10 gene expression [22–28], we examined the consequence of Wogonin treatment on them. Comparing to intact Foxp3, c-maf, JunB, Gata3, Egr2, Ahr, and Batf, Hif-1*α* was significantly abrogated. Hif-1*α* was demonstrated in driving IL-10 expression in CD1d^hi^CD5^+^ B cells [28]; this report echoed our conclusions. Meanwhile, Hif-1*α* induction was proved to depend on the STAT3/ERK signaling pathway.

In brief, on a different model, we showed that Wogonin treatment suppressed Hif-1*α* expression, STAT3/ERK signaling, and IL-10 secretion from B cells; this suggested novel molecular and cellular mechanism of Wogonin in B cells and their potential effect on immunomodulation.

## 5. Conclusions

Our study suggests that Wogonin may inhibit IL-10 production from B cells via modulating Hif-1*α* and the ERK, STAT3 signaling pathway, which provides novel and potential molecular targets of Wogonin in B cells and helps us further understand its mechanism and function in immune regulation, potentially improving its clinical application in the future.

## Figures and Tables

**Table 1:** The antibodies used in flow cytometry.

Antibody name	Supplier	Fluorochrome	Clone
CD19	BioLegend	Percp-cy5.5	6D5
MHC class II	BD	FITC/percp-cy5.5	M5/114.15.2
CD5	BioLegend	PEcy7	53-7.3
CD38	BD	FITC	90/CD38
CD80	BD	PE	16-10A1
CD86	BioLegend	APC-cy7	GL-1
CD21	BD	APC	7G6
CD24	BD	FITC	M1/69
CD23	BD	PE	B3B4
IgM	BD	APC	R6-60.2
IgD	BD	PE	11-26c.2a
IL-10	BioLegend	PE	JES5-16E3

**Table 2:** Sequence of primers used for RT-PCR.

Gene	5′ to 3′
Gata3	F: CTCGGCCATTCGTACATGGAA
	R: GGATACCTCTGCACCGTAGC
Batf	F: CTGGCAAACAGCACTCATCTG
	R: GGGTGTCGGCTTTCTGTGTC
c-maf	F: GGAGACCGACCGCATCATC
	R: TCATCCAGTAGTAGTAGTCTTCCAGG
Ahr	F: AGCCGGTGCAGAAAACAGTAA
	R: AGGCGGTCTAACTCTGTGTTC
Egr2	F: GCCAAGGCCGTAGACAAAATC
	R: CCACTCCGTTCATCTGGTCA
Foxp3	F: CCCATCCCCAGGAGTCTTG
	R: ACCATGACTAGGGGCACTGTA
JunB	F: TCACGACGACTCTTACGCAG
	R: CCTTGAGACCCCGATAGGGA
Hif-1*α*	F: ACCTTCATCGGAAACTCCAAAG
	R: CTGGTAGGCTGGGAAAAGTTAGG
GAPDH	F: TCAATGAAGGGGTCGTTGAT
	R: CGTCCCGTAHACAAAATGGT

## Data Availability

1. The [original] data used to support the findings of this study are included within the article. 2. The [original] data used to support the findings of this study are included within the supplementary file.

## Supplementary Materials

Supplementary Materials The supplementary data includes the assessment of B cell purity and the viability and phenotypes of B cell in different cultural conditions. Figure S1: assessment of the purity of CD19^+^ B cells. Figure S2: effect of Wogonin on the viability of LPS-treated B cells. The purified CD19^+^ cells from the spleen were stimulated with or without LPS in the presence or absence of Wogonin (0, 12.5, 25, 50, and 100 *μ*M) for 6 h, 12 h, 24 h, and 48 h. Cells were harvested and the viability was assayed by a CCK8 kit. Figure S3: effect of Wogonin on the surface molecules of B cell. CD19^+^ cells were cultured for 24 h and 48 h in the presence or absence of Wogonin (12.5 *μ*M). (a, b) The expression levels of CD5, CD24, CD21, CD38, CD23, MHCII, IgM, IgD, CD80, and CD86 were measured by FACS at 24 h and 48 h, respectively. (c) Quantification of B cell surface molecules. Data are expressed as mean values ± SD of three independent experiments. ns: no significance.
