# Supplementary material for: Wogonin Suppresses IL-10 Production in B Cells via STAT3 and ERK Signaling Pathway
Source: J Immunol Res. 2020 Jun 1;2020:3032425. doi: 10.1155/2020/3032425 (PMC7285295; doi:10.1155/2020/3032425)
Supplement: Supplementary Materials — The supplementary data includes the assessment of B cell purity and the viability and phenotypes of B cell in different cultural conditions. Figure S1: assessment of the purity of CD19+ B cells. Figure S2: effect of Wogonin on the viability of LPS-treated B cells. The purified CD19+ cells from the spleen were stimulated with or without LPS in the presence or absence of Wogonin (0, 12.5, 25, 50, and 100 μM) for 6 h, 12 h, 24 h, and 48 h. Cells were harvested and the viability was assayed by a CCK8 kit. Figure S3: effect of Wogonin on the surface molecules of B cell. CD19+ cells were cultured for 24 h and 48 h in the presence or absence of Wogonin (12.5 μM). (a, b) The expression levels of CD5, CD24, CD21, CD38, CD23, MHCII, IgM, IgD, CD80, and CD86 were measured by FACS at 24 h and 48 h, respectively. (c) Quantification of B cell surface molecules. Data are expressed as mean values ± SD of three independent experiments. ns: no significance. [file 3032425.f1.pdf]

Supplementary Materials

Figure S1: Assessment of the purity of $CD19^{+}$B cells.

Figure S2: Effect of wogonin on the viability of LPS-treated B cells. The purified $CD19^{+}$ cells from spleen were stimulated with or without LPS in the presence or absence of wogonin (0, 12.5, 25, 50, 100 μM) for 6 h, 12 h, 24 h and 48 h. Cells were harvasted and the viability was assayed by a CCK8 kit.

Figure S3: Effect of wogonin on the surface molecules of B cell. $CD19^{+}$ cells were cultured for 24 h and 48 h in the presence or absence wogonin (12.5 μM). (a, b) The expression levels of CD5, CD24, CD21, CD38, CD23, MHCII, IgM, IgD, CD80 and CD86 were measured by FACS at 24 h and 48 h, respectively. (c) Quantification of B cell surface molecules. Data are expressed as mean ± SD of three independent experiments. ns, no significance.

Figure S1

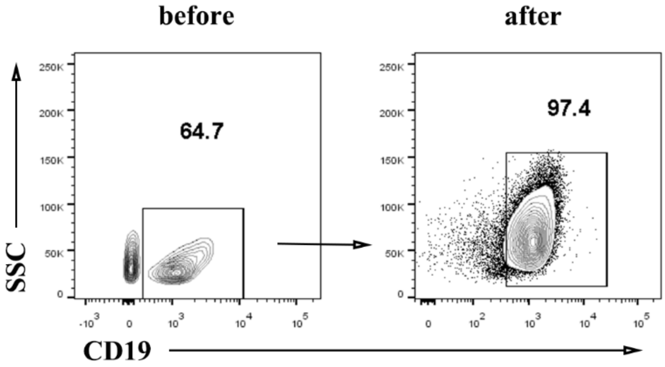

Figure S2

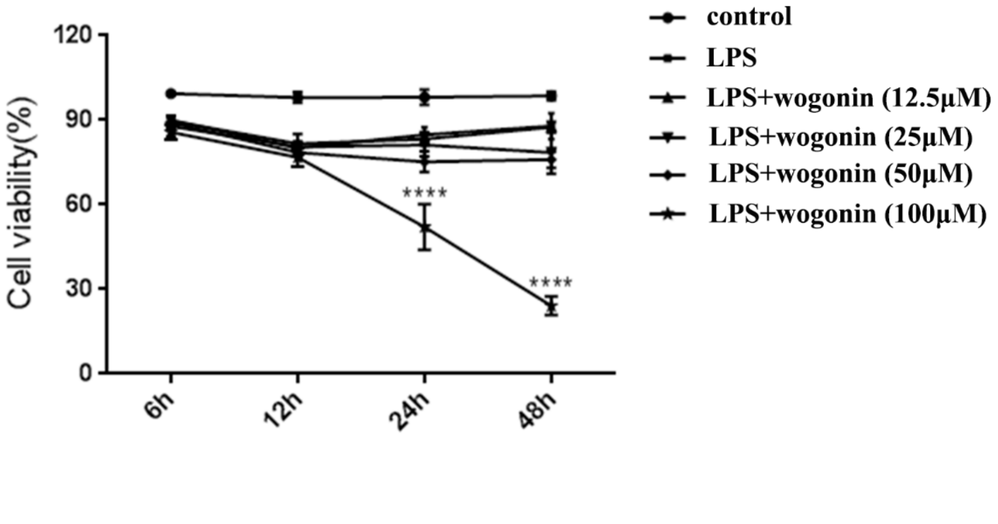

Figure S3

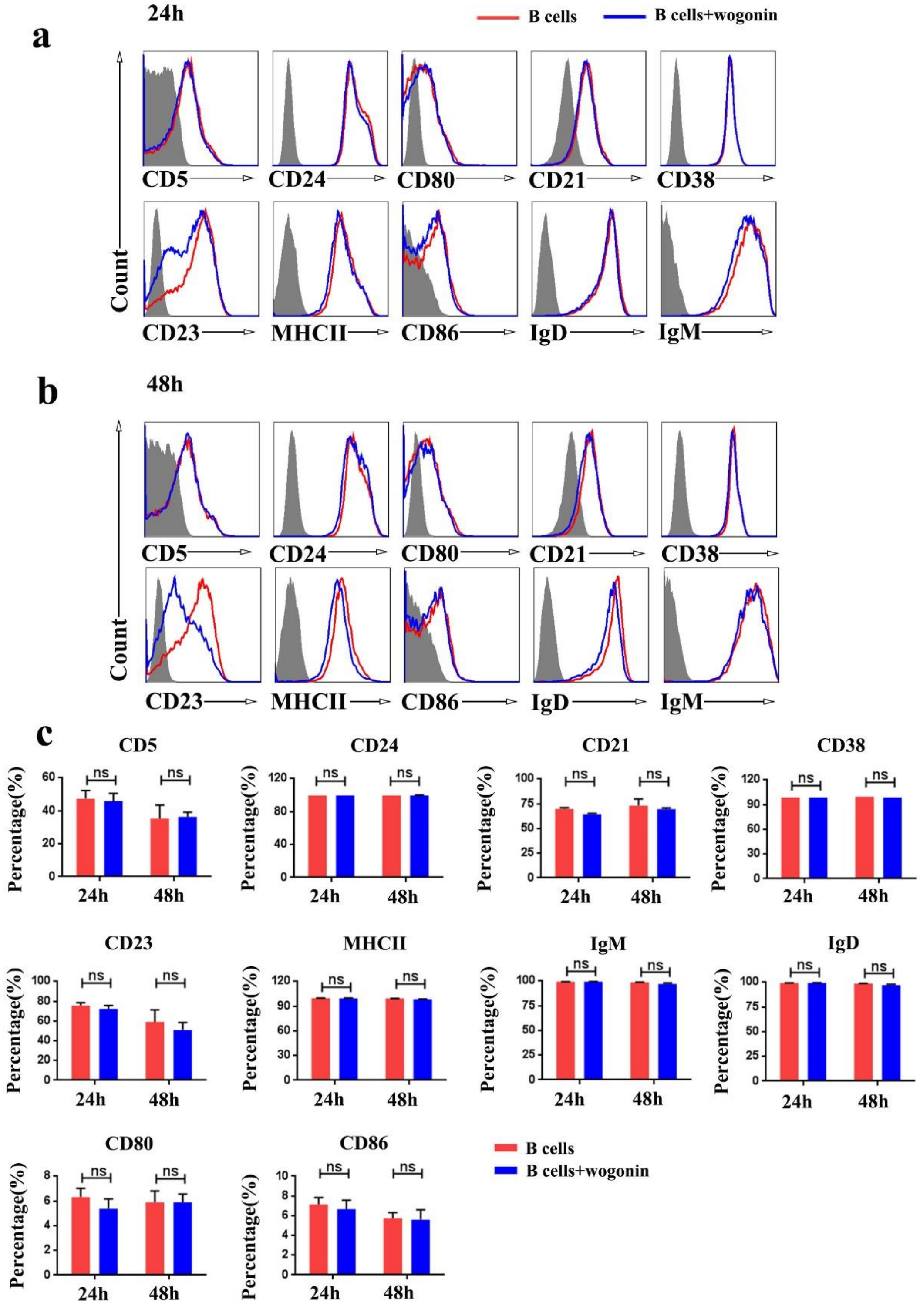
a
24h
B cells
B cells+wogonin
Count
CD5
CD24
CD80
CD21
CD38
CD23
MHCII
CD86
IgD
IgM
b
48h
c
CD5
CD24
CD21
CD38
CD23
MHCII
IgM
IgD
CD80
CD86
Percentage(%)
ns
24h
48h
